# Data vignettes for the application of response surface models in drug combination analysis

**DOI:** 10.1016/j.dib.2021.107400

**Published:** 2021-09-20

**Authors:** Nathaniel R. Twarog, Nancy E. Martinez, Jessica Gartrell, Jia Xie, Christopher L. Tinkle, Anang A. Shelat

**Affiliations:** a Department of Chemical Biology and Therapeutics, St. Jude Children's Research Hospital, Memphis, TN, United States; b Department of Radiation Oncology, St. Jude Children's Research Hospital, Memphis, TN, United States

**Keywords:** Combination therapy, Synergy, Response surface models

## Abstract

This data set contains the data used in Twarog et al. (2021) to examine the robustness and utility of response surface models in drug combination analysis. It includes simulated experimental data for the evaluation of traditional index methods, as well as a processed library of interaction metrics evaluated on the Merck OncoPolyPharmacology Screen (O'Neil et al., 2016), the scripts used to implement those metrics on all tested combinations in that screen, and scripts to evaluate the performance of those metrics in comparison with real-world mechanistic classifications. Finally, the data set includes data from several published and unpublished drug combination experiments, and scripts which allow the analyses of those experiments to be replicated and applied to new data.

## Specifications Table

**Table:** 

Subject	Computational Biology, Pharmacology
Specific subject area	Quantitative analysis of experimental combination therapy data
Type of data	Table Script
How data were acquired	Data were collected from previous publications and pre-processed, or simulated in the R computing environment
Data format	Pre-processed Analyzed
Parameters for data collection	Data were selected as deemed appropriate for the examination of response surface methods in combination analysis
Description of data collection	A collection of real-world and simulated drug combination experiments
Data source location	O'Neil, J., et al., An Unbiased Oncology Compound Screen to Identify Novel Combination Strategies. Mol Cancer Ther, 2016. 15(6): p. 1155–62. Weinstein, Z.B., et al., Modeling the impact of drug interactions on therapeutic selectivity. Nat Commun, 2018. 9(1): p. 3452. O'Brien, B., S. Chaturvedi, and V. Chaturvedi, *In Vitro* Evaluation of Antifungal Drug Combinations against Multidrug-Resistant Candida auris Isolates from New York Outbreak. Antimicrobial Agents and Chemotherapy, 2020. 64(4): p. e02195–19. Tan, X., et al., Systematic identification of synergistic drug pairs targeting HIV. Nat Biotechnol, 2012. 30(11): p. 1125–30. Butts, A., et al., A Systematic Screen Reveals a Diverse Collection of Medications That Induce Antifungal Resistance in Candida Species. Antimicrob Agents Chemother, 2019. 63(5), p. e00054–19.
Data accessibility	Repository name: Data Vignettes for the Application of Response Surface Models in Drug Combination Analysis Direct URL to data: https://data.mendeley.com/datasets/znpbzzvb7t/1
Related research article	N.R. Twarog, et al., Using response surface models to analyze drug combinations, Drug Discov Today, 2021.

## Value of the Data

• These data encompass a wide range of real and simulated combination experiment outcomes and modalities that allow for an in-depth examination of combination analysis techniques.
• Researchers developing methods for combination analysis or evaluation and researchers searching for the best existing analysis method can use these data as a template for their own research.
• The scripts included with this dataset can be adapted to new data so that researchers can replicate the analysis with their own experimental data.
• Examination of the results of simulated and real-world analyses allows researchers to determine what method is most useful for them.

## 1 Data Description

These data files encompass several demonstrations of the utility and efficacy of combination evaluation using response surface models such the Bivariate Response to Additive Interacting Doses, or BRAID model [1], the universal response surface approach, or URSA model [2], and the multidimensional synergy of combinations, or MuSyC model [3].

The first directory, Data01_Bias, contains no data, but instead R scripts which generate simulated experimental data based on a preset random seed.

The second directory, Data02_MerckOPPS is the largest and most complex, as it is the most in-depth examination of the various metrics. Most of the data is found in the subfolder RawData, which contains pre-processed tabular single agent data for all compounds and cell lines from the Merck OncoPolyPharmacology screen (OPPS) [4] in the file “AllSings.txt”, and pre-processed tabular combination data for all combinations and cell lines in the file “AllCombs.txt”. For easier processing these data files have also been broken into 32 blocks, each of which contains the relevant data for a distinct set of drug combinations in all the cell lines in which they were tested. The RawData folder also contains a classification of 32 of the 38 compounds into sixteen mechanistic classes in the file “Mechanisms_submit.txt”. The folder “AnalysisResults” contains two data files, with the best-fitting dose-response parameters for all single-agent experiments in the file “MerckOPPS_AllDRFits.txt”, and the best fit BRAID response surface parameters for all combination experiments, as well as estimates of the BRAID index of achievable efficacy (IAE), combination index, and an early version of the Bliss volume metric in the file “MerckOPPS_AllCMFits.txt”. The subfolder “Blocks” also contains these fits and metrics broken up by drug combination block. The folder “AnalysisScripts” contains two R scripts, “BreakBlocks.R” and “FitBlocks.R”, which can be used to replicate breaking the original raw data into blocks and performing the dose-response and response-surface fitting that produced the results in “AnalysisResults”; the text file “AnalysisSteps.txt” describes how these scripts can be run. The folder “ExtendedAnalysisResults” contains a data file called “MerckOPPS_AllExtendedMetrics.txt” with an even broader range of interaction metrics, including eight volumetric metrics based on Vlot et al. [5], the alpha parameter of URSA [2] and the three MuSyC interaction parameters [3]. The subfolder “Blocks” contains metric files broken up by drug combination block as well as the complete set of best fitting URSA and MuSyC response surface parameters, also broken up by block. This folder also contains two R data files, “MerckOPPS_CorrSimliarities.rda” and “MerckOPPS_CosineSimilarities.rda”, containing the results of correlation and cosine-based similarity analyses of 32 of the 38 drugs tested. The folder “ExtendedAnalysisScripts” contains three R scripts used to generate these extended results, including: “ExtendedFitBlocks.R”, which calculates all extended metrics; “ClusterAllMetrics.R”, which calculates the similarity between compounds imposed by each interaction metric using both cosine and correlation similarity; and “EvaluateClusters.R”, which evaluates the clustering induced by each similarity metric using the adjusted Rand index. This folder also contains two files, “fittingFunctions.R” and “clusterfuncs.R” which contain R functions used by these scripts.

The third directory, Data03_TherapeuticWindows, contains four data files from Weinstein et al. [6] containing the raw measurements of activity of two drug combinations – pentamidine (Pen) vs. staurosporine (Sta) and methyl-methanosulfate (MMS) vs. rapamycin (Rap) – in *S cerevisiae* and *C. albicans*. The subfolder “albRAW” contains the raw data measuring the effect of methyl-methanosulfate with rapamycin (“MMS-Rap.txt”) and the effect of pentamidine with staurosporine (“Pen-Sta.txt”) in the *C. albicans*. The subfolder “cerRAW” contains the corresponding files measuring effects in *S. cerevisiae*. Each file contains a 64 column matrix of numbers, which corresponds to OD595 readings for one drug‐drug interaction. Rows correspond to different time points with 15 min intervals. Columns correspond to the 8 × 8 matrix of drug concentration combinations. Finally, the folder contains an R script, “Data03_Script01_TherapeuticWindows.R” (along with a file containing necessary R functions, “fitBraid8Par.R”) that extracts the activity of all combinations from these files, fits them with the BRAID response surface model, and plots the resulting fit response surfaces.

The fourth directory, Data04_AlternateEndpoints, contains three preprocessed data files from O'Brien et al. [7] containing the single agent minimum inhibitory concentration (MIC) results (“Data04_Raw01_OBrienEtAl_SingleAgent.txt”), drug combination MIC results (“Data04_Raw02_OBrienEtAl_Combinations.txt”), and compound information (“Data04_Raw03_OBrienEtAl_Compounds.txt”) from a drug screen testing for inhibition of an isolate of *C. auris*. The single agent and combination data files contain one record for each dose or dose pair tested, with a MIC value of 1 for those dose or dose pairs that lie at or above the measured MIC, and a value of 0 for those that lie below it. The directory also contains a fourth file (“Data04_Raw04_ClonogenicCounts.txt”) containing colony counts of the desmoplastic small round cell cancer line JN-DSRCT-1 exposed to combinations of varying doses of radiation and the PARP inhibitor talazoparib. The R script file “Data04_Script01_AlternateEndpoints.R” extracts the data from these four files and performs two response surface fits, reporting the results.

The fifth directory, Data05_ResponseVolumes, contains the raw assay data from an in vitro viability experiment testing the three-drug combination of rolipram, prednisolone, and forskolin in the acute lymphoblastic leukemia cell line 697, in a file called “Data05_Raw01_ThreeDrugCombination.txt”. The R script file “Data05_Script01_ResponseVolumes.R” extracts this data and fits it with a three-drug extended BRAID model.

The sixth directory, Data06_AtypicalSurfaces, contains a pre-processed tabular file (“Data06_Raw01_TanEtAl_HIVcombination.txt”) containing the assay results for docosanol and roxithromycin in a human immunodeficiency virus (HIV) screen reported by Tan et al. [8]. It also contains a pre-processed tabular data file (“Data06_Raw02_ButtsEtAl_Antifungal.txt”) containing the assay results for penbutolol and fluconazole in an antifungal screen reported by Butts et al. [9]. The R script file “Data06_Script01_AtypicalSurfaces.R” extracts the data from these two files, fits them with a traditional BRAID surface, as well as a transformed oppositional or protective BRAID surface, and reports all fit results.

## 2 Experimental Design, Materials and Methods

For most data, the methods used to acquire the data can be found in the original relevant publication. The methods used to analyze the data are described in the main text and supplemental text of Twarog et al. [10]. We have included all R scripts used to perform the relevant analyses, which if run will output the results reported in our manuscript [10].

## CRediT authorship contribution statement

**Nathaniel R. Twarog:** Conceptualization, Methodology, Software, Data curation, Writing – original draft, Writing – review & editing. **Nancy E. Martinez:** Data curation, Investigation. **Jessica Gartrell:** Data curation, Investigation. **Jia Xie:** Data curation, Investigation. **Christopher L. Tinkle:** Conceptualization, Supervision. **Anang A. Shelat:** Conceptualization, Methodology, Writing – original draft, Writing – review & editing, Supervision.

## Declaration of Competing Interest

The authors declare that they have no known competing financial interests or personal relationships which have or could be perceived to have influenced the work reported in this article. This manuscript adheres to the Ethics in publishing standards (https://www.elsevier.com/about/policies/publishing-ethics#Authors).
